# CNS germinomas are characterized by global demethylation, chromosomal instability and mutational activation of the Kit-, Ras/Raf/Erk- and Akt-pathways

**DOI:** 10.18632/oncotarget.10392

**Published:** 2016-07-04

**Authors:** Simone Laura Schulte, Andreas Waha, Barbara Steiger, Dorota Denkhaus, Evelyn Dörner, Gabriele Calaminus, Ivo Leuschner, Torsten Pietsch

**Affiliations:** ^1^ Department of Neuropathology, University of Bonn Medical Center, Bonn, Germany; ^2^ Department of Pediatric Hematology/Oncology, University of Bonn Medical Center, Bonn, Germany; ^3^ Kiel Paediatric Tumor Registry, Department of Paediatric Pathology, University Hospital of Schleswig-Holstein, Campus Kiel, Germany

**Keywords:** germinoma, c-kit, ras, hypomethylation, genomics

## Abstract

CNS germinomas represent a unique germ cell tumor entity characterized by undifferentiated tumor cells and a high response rate to current treatment protocols. Limited information is available on their underlying genomic, epigenetic and biological alterations. We performed a genome-wide analysis of genomic copy number alterations in 49 CNS germinomas by molecular inversion profiling. In addition, CpG dinucleotide methylation was studied by immunohistochemistry for methylated cytosine residues. Mutational analysis was performed by resequencing of candidate genes including *KIT* and *RAS* family members. Ras/Erk and Akt pathway activation was analyzed by immunostaining with antibodies against phospho-Erk, phosho-Akt, phospho-mTOR and phospho-S6. All germinomas coexpressed Oct4 and Kit but showed an extensive global DNA demethylation compared to other tumors and normal tissues. Molecular inversion profiling showed predominant genomic instability in all tumors with a high frequency of regional gains and losses including high level gene amplifications. Activating mutations of *KIT* exons 11, 13, and 17 as well as a case with genomic *KIT* amplification and activating mutations or amplifications of *RAS* gene family members including *KRAS*, *NRAS* and *RRAS2* indicated mutational activation of crucial signaling pathways. Co-activation of Ras/Erk and Akt pathways was present in 83% of germinomas. These data suggest that CNS germinoma cells display a demethylated nuclear DNA similar to primordial germ cells in early development. This finding has a striking coincidence with extensive genomic instability. In addition, mutational activation of Kit-, Ras/Raf/Erk- and Akt- pathways indicate the biological importance of these pathways and their components as potential targets for therapy.

## INTRODUCTION

Intracranial germ cell tumors (iGCTs) occur mostly in children and young adults. They account for 3-5% of pediatric CNS tumors with a five- to eightfold higher incidence in East Asia than in Western countries and affect more male than female patients (3:1) [1–3]. iGCTs are classified into pure germinomas and nongerminomatous germ cell tumors (NGGCT), including teratoma, embryonal carcinoma, yolk sac tumor, choriocarcinoma and malignant mixed iGCTs compound of teratomas and one or more of the other histologies. Pure germinoma is the most common subtype followed by mixed iGCTs. An important prognostic factor is the tumor's histology. Pure germinomas are very sensitive to radio- and chemotherapy leading to a 5-year survival rate of more than 90%. In contrast, mixed iGCTs vary in radio- and chemotherapeutic sensitivity; with 5-year survival rates less than 60% [4] for multi-modal therapy. Even though survival rates for germinomas are high long-term outcomes and late effects of radio- and chemotherapy including secondary neoplasms, endocrine deficiencies and strokes, demonstrate that further research with regard to targeted therapy is necessary [5].

The cellular origin of iGCTs is still unclear and controversial. For a long time, a popular hypothesis suggested that all GCTs originate from primordial germ cells (PGCs) migrating in the midline during development. Recent studies argue that iGCTs may develop from neural stem cells [6–8]. In addition, tumor biology underlying the pathogenesis of these tumors is also largely unknown and therefore a subject of current research. However, mutations and other alterations in the KIT/RAS- and AKT/mTOR pathway, as well as gains of chromosomes 12p or X are common biological/genetic alterations in iGCTs and seem to play a crucial role in the tumorigenic process [9–14] although their impact on survival is still unclear.

Gain-of-function-mutations of the tyrosine kinase KIT lead to a permanent pathological activation of this transmembrane protein, independent of binding its ligand stem cell factor (SCF) [15]. This results in an increased and steady stimulation of cell proliferation. A similar mechanism is described for *RAS* mutations, in particular *HRAS*, *KRAS* and *NRAS*. These small G-proteins play a central part in diverse signalling pathways and are activated by binding GTP. Point mutations in the active center often lead to an inability to bind GTPase which induces stimulation of cell growth [16].

Due to the fact that iGCTs are more frequent in East Asia than in the West, previous studies included mostly Asian patients. Our study is the first genetic analysis of a larger series of germinomas from Caucasian patients. Moreover, we provide immunohistochemical data on global DNA methylation and examined activation of ERK pathway to elaborate further information on the cell of origin, pathogenetic pathway(s) as well as potential novel therapeutic targets.

## RESULTS

### Clinical features

This study enrolled 55 patients with a median age at diagnosis of 17 years, ranging from 8 to 40 years (Table 1). There were 47 (85.5%) male and 8 (14.5%) female patients. Tumors included in this cohort predominantly affected the region of the pineal gland (31 of 55 [56.4%]) and the suprasellar region (11 of 55 [20%]). 6 germinomas (10.9%) revealed bifocal tumor location, 6 (10.9%) were located in other regions of the brain (pons, hypothalamus and lateral ventricle) and for 1 case (1.8%) information about the exact location was not available. For 33 cases survival data was available, revealing that no patient had metastasis, 6 showed subsequent events and 2 patients died. These 33 patients were all of Caucasian ethnicity. In addition, there was no indication of an Asian or Afroamerican ethnic background from the available data of the other cases. 54 tumors were diagnosed as pure germinomas, only 1 case was considered as mixed GCT with large germinoma contents and a teratoma component. All 55 cases showed c-Kit and Oct4 expression, which are recognized diagnostic criteria for germinomas.

**Table 1:** Clinical data, genetic alteration status and ERK-/ Akt/mTOR activation in 55 germinoma cases

Case No.	Histology	Age	Sex	Location	DNA hypomethylation	stable Genome	MIP amplification	c-Kit mutation	RAS mutation	ERK activation	Akt/mTOR activation	Event	Death
1	Germinoma	12	m	Suprasellar	yes	no	no	no	yes	yes	yes	no	no
2	Germinoma	11	f	bifocal	yes	no	no	yes	no	no	yes	yes	yes
3	Germinoma	12	m	Pinealis	yes	no	chr. 12p12	no	no	yes	yes	yes	yes
4	Germinoma	19	m	Pinealis	yes	no	no	no	no	yes	yes	yes	no
5	Germinoma	15	m	Pinealis	yes	no	no	no	yes	yes	yes	no	no
6	Germinoma	10	m	other	yes	no	no	no	no	yes	yes	no	no
7	Germinoma	15	f	Suprasellar	yes	no	chr. 12p12	no	no	yes	yes	no	no
8	Germinoma	13	f	Suprasellar	yes	no	no	no	no	no	yes	no	no
9	Germinoma	21	m	other	yes	no	no	no	no	yes	yes	n.a.	n.a.
10	Germinoma	14	m	Pinealis	yes	no	no	no	no	n.a.	yes	yes	no
11	Germinoma	31	m	other	yes	no	no	no	yes	yes	yes	no	no
12	Germinoma	17	m	bifocal	yes	no	chr. 11p15.2	no	no	yes	yes	no	no
13	Germinoma	11	m	Pinealis	yes	no	no	no	no	no	yes	no	no
14	Germinoma	13	f	Suprasellar	yes	no	chr. 12p12	no	no	yes	yes	n.a.	n.a.
15	Germinoma	17	m	bifocal	yes	no	chr. 12p12	no	no	yes	no	no	no
16	Germinoma	18	m	Pinealis	n.a.	no	no	no	yes	n.a.	n.a.	n.a.	n.a.
18	Germinoma	13	m	Suprasellar	yes	no	no	no	yes	yes	yes	no	no
19	Germinoma	13	m	Pinealis	yes	no	no	no	yes	yes	yes	no	no
20	Germinoma	15	f	Suprasellar	yes	no	no	yes	no	yes	yes	no	no
21	Germinoma	11	m	Pinealis	yes	no	no	no	yes	yes	yes	no	no
22	Germinoma	21	m	Pinealis	yes	no	no	no	no	yes	yes	n.a.	n.a.
23	Germinoma	24	m	Pinealis	yes	no	no	yes	no	yes	yes	n.a.	n.a.
24	Germinoma	18	m	Pinealis	yes	no	chr. 4q12	no	no	yes	yes	no	no
25	Germinoma	15	m	Pinealis	yes	no	no	no	yes	yes	yes	no	no
27	Germinoma	17	m	Pinealis	yes	no	no	no	no	yes	yes	no	no
28	Germinoma	11	m	Pinealis	yes	no	no	no	yes	yes	yes	no	no
29	Germinoma	14	m	bifocal	yes	no	chr. 12p12	no	no	yes	yes	no	no
30	Germinoma	17	m	Pinealis	yes	no	no	no	no	yes	yes	n.a.	n.a.
31	Germinoma	16	m	Pinealis	yes	no	no	no	no	yes	yes	no	no
33	Germinoma	16	m	Pinealis	yes	no	no	yes	no	yes	yes	no	no
34	Germinoma	24	m	Pinealis	yes	no	no	no	no	yes	yes	n.a.	n.a.
36	Germinoma	22	m	Pinealis	yes	no	no	no	no	yes	yes	n.a.	n.a.
37	Germinoma	40	m	Suprasellar	yes	no	no	no	no	yes	yes	n.a.	n.a.
38	Germinoma	18	m	other	yes	no	no	no	yes	yes	yes	n.a.	n.a.
39	Germinoma	20	m	Suprasellar	yes	no	no	yes	no	yes	yes	n.a.	n.a.
40	Germinoma	11	m	Suprasellar	yes	no	no	no	no	yes	yes	n.a.	n.a.
41	Germinoma	19	m	Pinealis	yes	no	no	no	no	yes	yes	no	no
42	mixed GCT (Germinoma with teratoma component)	11	m	other	yes	no	no	no	yes	yes	yes	no	no
43	Germinoma	10	m	bifocal	yes	no	no	yes	no	no	yes	no	no
44	Germinoma	17	m	Pinealis	yes	no	no	no	yes	yes	yes	n.a.	n.a.
45	Germinoma	19	f	bifocal	yes	no	no	no	no	yes	yes	no	no
46	Germinoma	8	f	Suprasellar	yes	no	no	no	no	yes	yes	no	no
47	Germinoma	8	m	Pinealis	yes	no	no	no	no	yes	yes	no	no
48	Germinoma	13	m	n.a.	yes	no	no	no	no	yes	yes	n.a.	n.a.
49	Germinoma	28	m	Pinealis	yes	no	no	no	no	no	no	n.a.	n.a.
50	Germinoma	29	m	Pinealis	yes	no	no	yes	no	yes	yes	n.a.	n.a.
51	Germinoma	27	m	Pinealis	yes	no	no	no	no	yes	yes	n.a.	n.a.
52	Germinoma	25	m	Pinealis	yes	no	no	yes	no	yes	yes	n.a.	n.a.
53	Germinoma	24	m	Pinealis	yes	n.a.	n.a.	n.a.	n.a.	yes	yes	n.a.	n.a.
54	Germinoma	20	m	Pinealis	yes	no	no	n.a.	n.a.	yes	yes	n.a.	n.a.
55	Germinoma	21	f	other	yes	n.a.	n.a.	n.a.	n.a.	yes	yes	no	no
17	Germinoma	34	m	Pinealis	yes	yes	no	no	no	no	no	n.a.	n.a.
26	Germinoma	19	m	Pinealis	yes	yes	no	no	no	yes	yes	n.a.	n.a.
32	Germinoma	12	m	Pinealis	yes	yes	no	no	no	yes	yes	no	no
35	Germinoma	13	m	Suprasellar	yes	yes	no	no	no	n.a.	yes	no	no

Germinoma 17, 26, 32 and 35 are listed at the end and marked grey, because they are excluded from MIP and mutation analysis due to their minimal content of tumor cells. Bifocal location stands for tumor occurrence in both pineal gland and suprasellar region.

**Figure 1:**
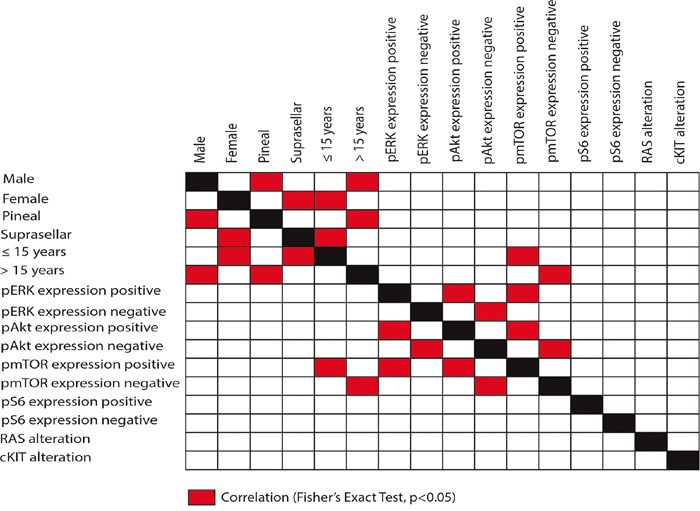
Correlation with clinico-pathological data All p-values refer to the Fisher's exact test. p-values < 0.05 were considered as statistically significant.

Most interestingly we discovered an association between female gender and age ≤ 15 years (p=0.004) as well as suprasellar tumor location (p=0.016). Conversely, male gender was associated with age >15 years (p=0.004) and pineal tumor location (p=0.023) (Figure 1). Survival data of 33 patients treated according the SIOP/HIT germ cell tumor protocols were available. Progression-free survival or overall survival did not correlate to the genetical or biological alterations evaluated in this study.

### Global hypomethylation of germinomas

**Figure 2:**
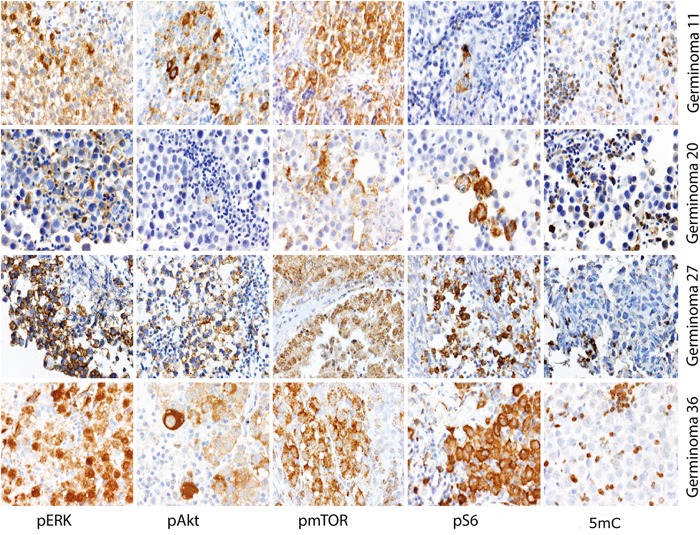
Representative staining results of pERK, pAkt, pmTOR, pS6 and 5mC All four cases revealed ERK and Akt/mTOR pathway activation and global demethylation.

Immunohistochemical staining against the 5-Methylcytosine (5mC) epitope of DNA was performed on 54 tumor specimens. All examined samples showed no immunoreactivity for 5mC in tumor cell nuclei, indicating that global DNA hypomethylation is the rule in germinomas (Figure 2). In contrast, peritumoral and tumor-infiltrating lymphocytes, which are characteristic of germinomas, showed strong nuclear staining for this epigenetic hallmark.

### MIP analysis

**Figure 3:**
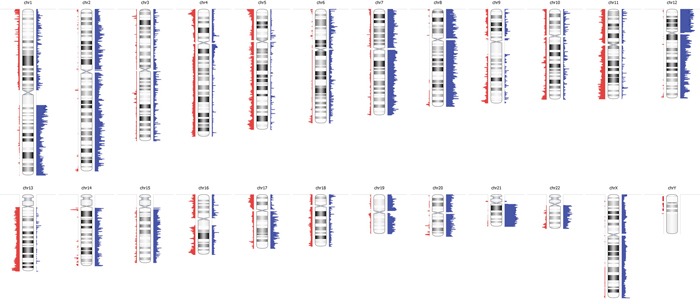
Virtual karyogram of 49 germinomas included in this study Chromosomal instability was detected by molecular inversion probe (MIP) arrays. Gains are indicated in blue and losses in red. Frequent gains of chromosomes 1q, 7, 8, 12p and 21q as well as losses of 5q, 9q, 11q, 13q, and 16p become obvious.

Molecular inversion probe analysis was performed for 53 germinomas for which DNA was available. 4 cases (7.5%) showed a stable genome but histologically, they consisted mostly of lymphocytes with only a minimal amount of germinoma cells. For this reason we excluded them from data analysis. We detected extensive genetic alterations and chromosomal instability in all remaining 49 cases (92.5%) (Figure 3). Most frequently, germinomas showed gains on chromosomes 12p (82%), 21q (76%), 8 (67%), 1q (65%) and 7 (59%). Chromosomal losses were less frequent than gains and were most commonly related to chromosomes 13q (45%), 11q (41%), 5q, 9q (both 39%), and 16p (37%).

**Figure 4:**
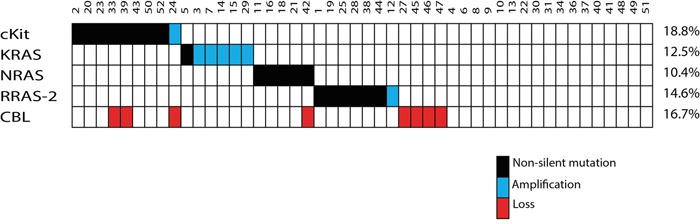
Summary of the somatic events Each mutation or alteration found in *KIT*, *KRAS*, *NRAS* and *RRAS-2* is a mutually exclusive event in the affected germinoma.

Of note, amplification at 12p12, involving *KRAS*, was detected in 5 cases (10.2%) representing the most frequent amplification. In addition, amplification at 4q12 and 11p15.2, affecting *KIT* and *RRAS-2*, were identified in one case (2%) each (Figure 4). Loss of *CBL* at chromosome 11q23.3 was found in 8 cases (16.3%).

**Figure 5:**
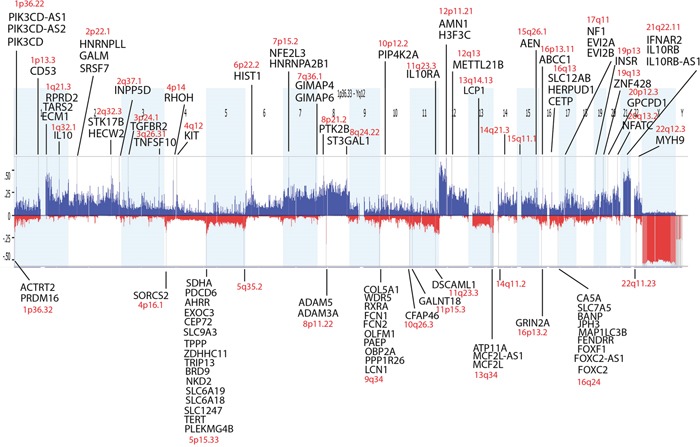
Statistically significant frequent copy number variations in germinomas analyzed by GISTIC Germinomas reveal a high level of genomic instability. P-values ≤ 0.001 were defined as statistically significant. Protein-coding genes in the affected regions are listed.

GISTIC analysis was used to distinguish significant chromosomal aberrations from random background and revealed a substantial number of copy number (CN) alterations in germinomas. 33 CN gains and 14 CN losses were detected within the germinoma genome by setting the significance cut-off to p≤0.001 (Figure 5). 94% of gains and 79% of losses included protein-coding regions. Remarkably, CN gains affected the *IL10* (Interleukin-10) gene and genes encoding its receptors *IL10RA, IL10RB* and *IL10RB-AS1* at chromosomes 1q32.1, 11q23.3 and 21q22.11. Moreover, chromosome 4q12, including *KIT*, showed also significant CN gains (Table 2).

**Table 2:** Protein-coding genes affected by significant CN gains and losses

	Region	Gene	Name
**Gains**			
	*Chr. 1p36.22*	PIK3CD-AS1	PIK3CD antisense RNA 1
		PIK3CD-AS2	PIK3CD antisense RNA 2
		PIK3CD	Phosphatidylinositol-4,5-bisphosphate 3-kinase, catalytic subunit delta
	*Chr. 1p13.3*	CD53	CD53 molecule
	*Chr. 1q21.3*	RPRD2	Regulation of nuclear pre-mRNA domain containing 2
		TARS2	Threonyl-tRNA synthetase 2, mitochondrial (putative)
		ECM1	Extracellular matrix protein 1
	*Chr. 1q32.1*	IL10	Interleukin 10
	*Chr. 2p22.1*	HNRNPLL	Heterogeneous nuclear ribonucleoprotein L-like
		GALM	Galactose mutarotase (aldose 1-epimerase)
		SRSF7	Serine/arginine-rich splicing factor 7
	*Chr. 2q32.3*	STK17B	Serine/threonine kinase 17b
		HECW2	HECT, C2 and WW domain containing E3 ubiquitin protein ligase 2
	*Chr. 2q37.1*	INPP5D	Inositol polyphosphate-5-phosphatase D
	*Chr. 3p24.1*	TGFBR2	Transforming growth factor, beta receptor II (70/80kDa)
	*Chr. 3q26.31*	TNFSF10	Tumor necrosis factor (ligand) superfamily, member 10
	*Chr. 4p14*	RHOH	Ras homolog family member H
	*Chr. 4q12*	KIT	v-kit Hardy-Zuckerman 4 feline sarcoma viral oncogene homolog
	*Chr. 6p22.2*	HIST1	Histone cluster 1
	*Chr. 7p15.2*	NFE2L3	Nuclear factor, erythroid 2-like 3
		HNRNPA2B1	Heterogeneous nuclear ribonucleoprotein A2/B1
	*Chr. 7q36.1*	GIMAP4	GTPase, IMAP family member 4
		GIMAP6	GTPase, IMAP family member 6
	*Chr. 8p21.2*	PTK2B	Protein tyrosine kinase 2 beta
	*Chr. 8q24.22*	ST3GAL1	ST3 beta-galactoside alpha-2,3-sialyltransferase 1
	*Chr. 10p12.2*	PIP4K2A	Phosphatidylinositol-5-phosphate 4-kinase, type II, alpha
	*Chr. 11q23.3*	IL10RA	Interleukin 10 receptor, alpha
	*Chr. 12p11.21*	AMN1	Antagonist of mitotic exit network 1 homolog
		H3F3C	H3 histone, family 3C
	*Chr. 12q13*	METTL21B	Methyltransferase like 21B
	*Chr. 13q14.13*	LCP1	Lymphocyte cytosolic protein 1 (L-plastin)
	*Chr. 15q26.1*	AEN	Apoptosis enhancing nuclease
	*Chr. 16p13.11*	ABCC1	ATP-binding cassette, sub-family C (CFTR/MRP), member 1
	*Chr. 16q13*	SLC12AB	Solute carrier family 12 (sodium/chloride transporter), member 3
		HERPUD1	Homocysteine-inducible, endoplasmic reticulum stress-inducible, ubiquitin-like domain member 1
		CETP	Cholesteryl ester transfer protein, plasma
	*Chr. 17q11*	NF1	Neurofibromin 1
		EVI2A	Exotropic viral integration site 2A
		EVI2B	Exotropic viral integration site 2B
	*Chr. 19p13*	INSR	Insulin receptor
	*Chr. 19q13*	ZNF428	Zinc finger protein 428
	*Chr. 20p12.3*	GPCPD1	Glycerophosphocholine phosphodiesterase 1
	*Chr. 20q13.2*	NFATC	Nuclear factor of activated T-cells, cytoplasmic, calcineurin-dependent 2
	*Chr. 21q22.11*	IFNAR2	Interferon (alpha, beta and omega) receptor 2
		IL10RB	Interleukin 10 receptor, beta
		IL10RB-AS1	IL10RB antisense RNA 1 (head to head)
	*Chr. 22q12.3*	MYH9	Myosin, heavy chain 9, non-muscle
**Losses**			
	*Chr. 1p36.32*	ACTRT2	Actin-related protein T2
		PRDM16	PR domain containing 16
	*Chr. 4p16.1*	SORCS2	Sortilin-related VPS10 domain containing receptor 2
	*Chr. 5p15.33*	SDHA	Succinate dehydrogenase complex, subunit A, flavorprotein (Fp)
		PDCD6	Programmed cell death 6
		AHRR	Aryl-hydrocarbon receptor repressor
		EXOC3	Exocyst complex component 3
		CEP72	Centrosomal protein 72kDa
		SLC9A3	Solute carrier family 9, subfamily A (NHE3, cation proton antiporter 3), member 3
		TPPP	Tubulin polymerization promotin protein
		ZDHHC11	Zinc finger, DHHC-type containing 11
		TRIP13	Thyroid hormone receptor interactor 13
		BRD9	Bromodomain containing 9
		NKD2	Naked cuticle homolog 2 (Drosophila)
		SLC6A18	Solute carrier family 6 (neutral amino acid transporter), member 18
		SLC6A19	Solute carrier family 6 (neutral amino acid transporter), member 19
		SLC1247	Solute carrier family 12 (potassium/chloride trasporter), member 7
		TERT	Telomerase reverse transcriptase
		PLEKHG4B	Pleckstrin homology domain containing, family G (with RhoGef domain) member 4B
	*Chr. 8p11.22*	ADAM3A	ADAM metallopeptidase domain 3A (pseudogene)
		ADAM5	ADAM metallopeptidase domain 5 (pseudogene)
	*Chr. 9q34*	COL5A1	Collagen, type V, alpha 1
		WDR5	WD repeat domain 5
		RXRA	Retinoid X receptor, alpha
		FCN1	Ficolin (collagen/fibrinogen domain containing) 1
		FCN2	Ficolin (collagen/fibrinogen domain containing lectin) 2
		OLFM1	Olfactomedin 1
		PAEP	Progestogen-associated endometrial protein
		OBP2A	Odorant binding protein 2A
		PPP1R26	Protein phosphatase 1, regulatory subunit 26
		LCN1	Lipocalin 1
	*Chr. 10q26.3*	CFAP46	Cilia and flagella associated protein 46
	*Chr. 11p15.3*	GALNT18	Polypeptide N-acetylgalactosaminyltransferase 18
	*Chr. 11q23.3*	DSCAML1	Down syndrome cell adhesion molecule like 1
	*Chr. 13q34*	ATP11A	ATPase, class VI, type 11A
		MCF2L-AS1	MCF2L antisense RNA 1
		MCF2L	MCF.2 cell line derived transforming sequence-like
	*Chr. 16p13.2*	GRIN2A	Glutamate receptor, ionotropic, N-methyl D-aspartate 2A
	*Chr. 16q24*	CA5A	Carbonic anhydrase VA, mitochondrial
		SLC7A5	Solute carrier family 7 (amino acid transporter light chain, L system), member 5
		BANP	BTG3 associated nuclear protein
		JPH3	Junctophilin 3
		MAP1LC3B	Microtubule-associated protein 1 light chain 3 beta
		FENDRR	FOXF1 adjacent non-coding developmental regulatory RNA
		FOXF1	Forkhead box F1
		FOXC2-AS1	FOXC2 antisense RNA 1

### Molecular analysis of *c-Kit* and *RAS* mutations in germinomas

We examined a total of 51 germinomas and 1 mixed GCT (germinoma and teratoma component) for mutations in *KIT* exons 11, 13, 17 and 18 as well as mutation hotspots in *HRAS*, *KRAS*, *NRAS* and *RRAS-2*. For the 4 cases (7.7%) showing a stable genome in MIP analysis, no mutations could be detected in *KIT* or *RAS*. Due to their minute amount of tumor cells they were excluded from the sequence data analysis.

**Figure 6:**
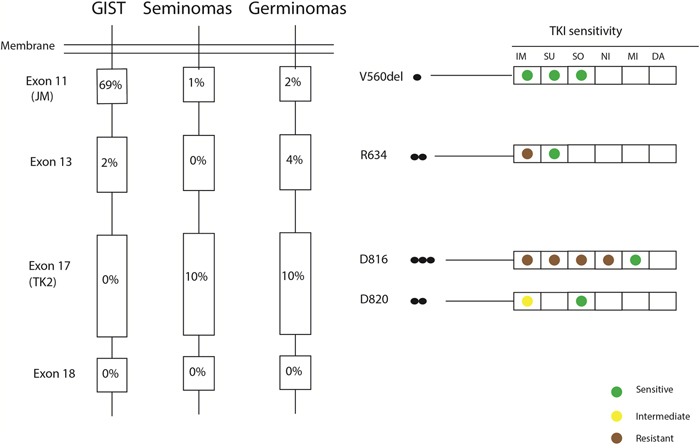
Somatic *KIT* mutations found in this germinoma cohort in comparison to reported *KIT* mutations in gastrointestinal stromal tumors (GISTs) and seminomas Black circles represent the number of cases harboring a given mutation. The functional domains concerned by mutations are juxtamembrane domain (JM) and tyrosine kinase II (TK2). Previously described tyrosine-kinase inhibitors (TKIs) [imatinib (IM), sunitinib (SU), sorafenib (SO), nilotinib (NI), midostaurin (MI) and dasatanib (DA)] and there activity against each mutation are shown on the right.

**Figure 7:**
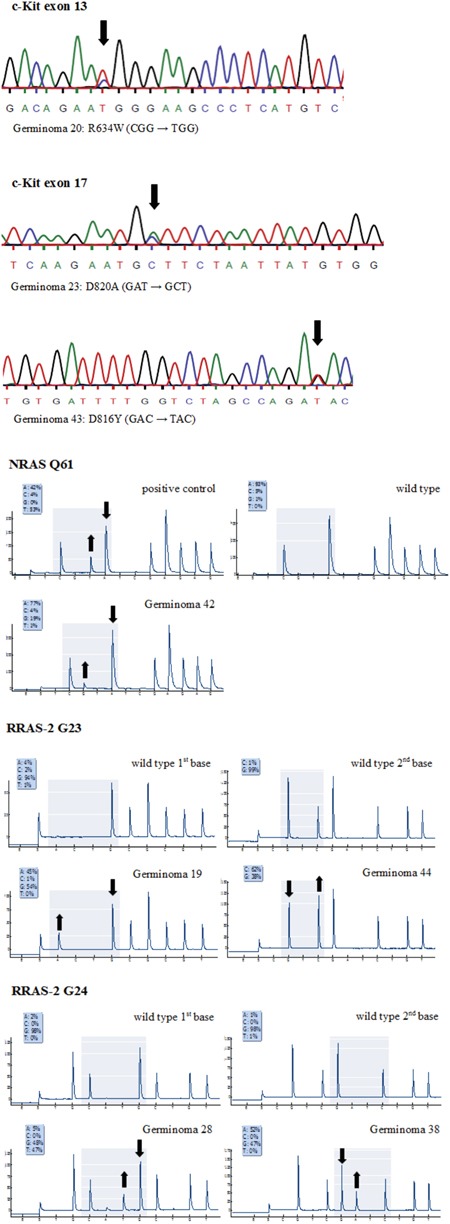
**a.** Representative *KIT* mutations detected by Sanger sequencing. **b.** Representative cases of *RAS* mutations detected by pyrosequencing analysis. Pyrograms are compared to wild type and/or positive control data. Significant peak increases and concomitant reductions in germinoma 42 [*NRAS* Q61R (CAA → CGA)], 19 [*RRAS-2* G23S (GGC → AGC)], 44 [*RRAS-2* G23A (GGC → GCC)], 28 [*RRAS-2* G24C (GGC → TGC)] and 38 [*RRAS-2* G24D (GGC → GAC)] expose mutations in these cases.

Sanger sequencing exposed non-silent *KIT* mutations in 8 cases (16.7%) (Figure 6). Most mutations affected tyrosine kinase II region (TK2) encoded by exon 17 in terms of point mutations in codons 816 (3/52) and 820 (2/52). In addition, one deletion of codon 560 in exon 11 and 2 point mutations in codon 634 of exon 13 were detected (representative sequencing results of *KIT* mutations are given in Figure 7a). No mutation in exon 18 of the *KIT* was observed.

**Figure 8:**
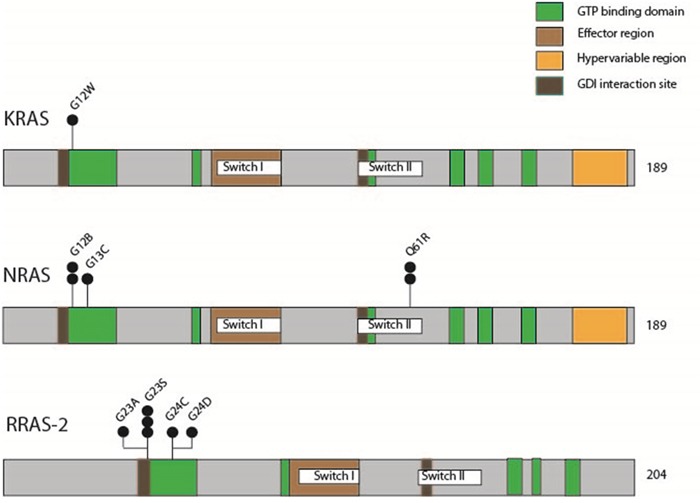
Schematic representation of mutations in *KRAS*, *NRAS* and *RRAS-2*, illustrating involvement of functional gene domains Black circles indicate the number of mutations identified.

Pyrosequencing analysis of the *RAS* mutation hotspots codon G12, G13 and Q61 in *HRAS*, *KRAS* and *NRAS* as well as their homologous parts codon G23, G24 and Q72 in *RRAS-2*, revealed 12 (25%) non-silent point mutations. Mainly, these point mutations affected the GTP binding domain (Figure 8).

Only one point mutation was seen in *KRAS* affecting codon G12. *NRAS* G12 and Q61 were each mutated in 2 tumors and *NRAS* G13 in 1. Most remarkably, no specimen revealed mutations in *HRAS*, but 6 (12.5%) germinomas showed point mutations in the RAS homolog *RRAS-2* of which 4 involved codon G23 and 2 involved codon G24 (representative sequencing results of *RAS* mutations are given in Figure 7b).

Altogether, genetic alterations were observed in 27 cases (56.3%) in *KIT* or *RAS* genes which were mutually exclusive (Figure 4). Comparison of *KIT*/*RAS* mutation status in germinomas and patient's age, sex and tumor location revealed no significant correlations (Figure 1).

### Immunohistochemical analysis of ERK- and Akt/mTOR-pathway

Immunohistochemical staining against pAkt, pmTOR, pS6 and pERK was performed on 54 GCTs including 53 pure germinomas and 1 mixed GCT (germinoma and teratoma component). Nuclear and cytoplasm staining of these proteins was considered positive.

pERK expression was observed in 46 (88.5%) tumors. Expression scores ranged from 0 to 300 (median, 102). 10 (19.2%) tumor samples showed strong staining for pERK. 24 (46.2%) tumor specimen revealed moderate staining whereas in 12 (23%) cases weak staining was found. No immunoreactivity for pERK was detected in 6 cases (11.5%).

45 (84.9%) tumor specimens showed expression of pAKT. Expression scores ranged from 0 to 300 (median, 101). Strong staining for pAkt was observed in 7 cases (13.2%). We detected moderate staining in 30 (56.6%) and weak staining in 8 (15.1%) samples. Negative staining was seen in 8 (15.1%) tumors.

pmTOR expression was detected in 45 (90%) tumor specimens. Expression scores ranged from 0 to 270 (median, 87). 3 (6%) cases were considered as strongly positive. 40 (80%) samples were rated moderate whereas 2 (4%) tumors disclosed weak staining. 5 (10%) germinomas revealed no immunoreactivity for the used pmTOR antibody.

**Figure 9:**
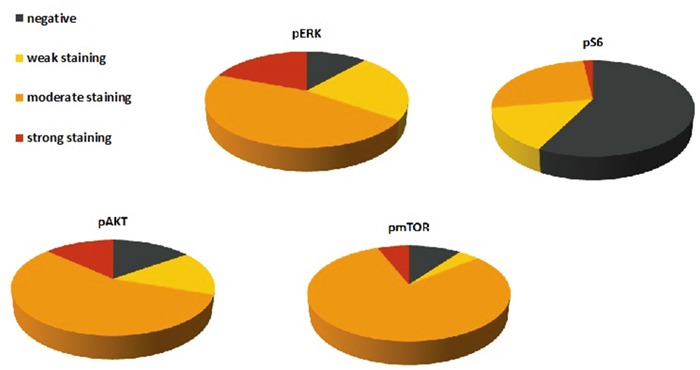
Protein expression of pERK, pAkt, pmTOR and pS6 in 54 germinomas Graphic presentation of distribution of negative, weak, moderate and strong staining against pERK, pAkt, pmTOR and pS6. pERK, pAkt and pmTOR were frequently expressed whereas pS6 showed more negative results.

In contrast to the other stainings, staining for pS6 was generally negative [31 tumors (57.4%)]. Only 23 (42.6%) samples revealed immunoreactivity for pS6. Expression scores ranged from 0 to 200 (median, 33). Strong staining was seen in only one (1.9%) case. 14 (25.9%) tumors showed moderate and 8 (14.8%) exhibited weak staining (Figure 9).

In seven cases, immunostaining could not be assessed because the samples contained only scant tumor cells in the presence of many lymphocytes (1 case for pAkt, 2 cases for pERK and 4 cases for pmTOR).

**Figure 10:**
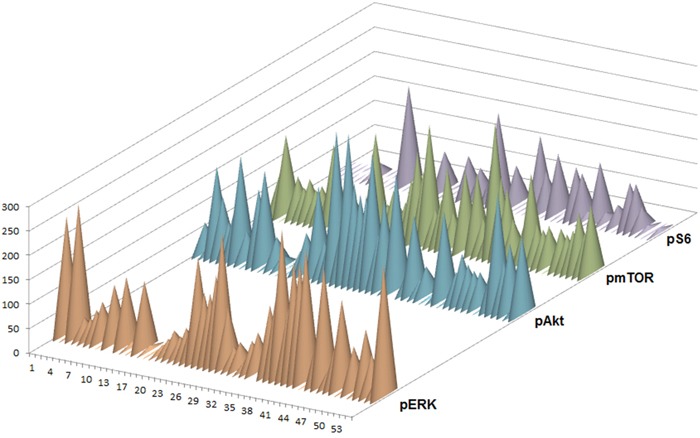
Semiquantitive analysis of pERK, pAkt, pmTOR and pS6 expression by IHC in 54 germinomas Staining was scored by multiplying intensity by percentage of stained cells. The result was a score between 0 and 300.

In summary, 88.5% of germinomas revealed an upregulation of the ERK pathway. Activation of AKT/mTOR pathway was seen in 94.4% of germinomas (illustration of IHC scores in Figure 10). Most remarkably, activation of both pathways was detected in 83.3% (representative staining results of cases with activations in both signal pathways are given in Figure 2).

## DISCUSSION

Our knowledge about the cellular origin of iGCTs is based on a limited number of studies. Analysis on mouse embryos showed that PGCs physiologically migrate in midline body structures during development, finally residing in the genital ridges [17]. Germ cells that remain in other areas usually die through reduction of stem cell factor signaling which leads to apoptosis of these PGCs [18]. In addition, Hoei-Hansen et al. [6] showed expression of genes associated with embryonic stem cell pluripotency like *PLAP*, *HCG*, *OCT-3/4*, *KIT, AP-2y* and *NANOG* in iGCTs. One hypothesis on the cellular origin of iGCTs is that they are derived from mismigrated PGCs. Alternatively, Tan and Scotting [7] suggested that transformation of endogenous neural stem cells by demethylation of the *OCT-4* promotor results in an upregulation of this gene which might trigger the formation of GCTs. Moreover, they assume that gene-specific methylation and additional genetic events, like *KIT* mutations in germinomas, might play a role in specification of these tumors. However, this hypothesis raises the question on the triggering mechanism involved in the demethylation of neural stem cells. In addition, this model does not explain why expression of c-Kit is not restricted to pure germinomas but also found as component in mixed GCTs [9].

Considering human germ cell development, global hypomethylation is an epigenetic hallmark in primordial germ cells (PGCs) accompanied by high c-Kit expression [19, 20]. We examined a large cohort of germinomas and showed that 100% of these revealed global hypomethylation and *c-Kit* expression in immunohistochemical staining, independently of *c-Kit* mutational status. In contrast to germinomas, studies revealed global methylation for yolk sac tumors [21] and non-seminomas [22], indicating a fundamental difference with germinomas in terms of lineage differentiation or cell of origin. Other brain tumor entities including different glioma entities, medulloblastomas and rhabdoid tumors of the CNS have been extensively characterised in the last years by genome-wide methylation profiling and did not show a similar demethylation phenotype [23–25]. Comparing the *KIT* mutational frequencies of exons 11, 13, 17 and 18 encoding different functional domains between germinomas, seminomas and gastrointestinal germ cell tumors (GISTs) showed strong similarities between seminomas and germinomas but a different distribution for GISTs [26, 27]. These results reinforce the genetic similitudes for seminomas and germinomas. Broader information on methylation status and *c-Kit* expression in larger collectives of NGGCTs could further help understanding differences of the pathogenesis in subtypes of iGCTs. Global DNA demethylation demonstrated by immunohistochemistry for 5mC may be a useful tool for the differential diagnosis of iGCTs. In addition, differences in therapeutic response between pure germinomas and NGGCTs could also be related to the tumors' methylation status.

In our study we showed that global hypomethylation of tumor DNA strikingly coincides with chromosomal instability in germinomas. Previously, Fukushima et al. [9] reported frequent gains of chromosomes 21q (66.7%), 1q (56.7%) and X (53.5%) and loss of chromosome 13q (53.3%) in a larger cohort of germinomas. These findings were confirmed by Wang et al. [14], describing chromosomes X (72.7%) and 21q (63.6%) as frequently amplified and 11q (45.5%), 13q and 17p (both 36.4%) frequently deleted in germinomas. Our findings reinforce these discoveries, showing frequent gains on chromosomes 12p (82%), 21q (76%), 8 (67%), 1q (65%) and 7 (59%) and losses on chromosomes 13q (45%), 11q (41%), 5q, 9q (both 39%), and 16p (37%) in pure germinomas. Of note, chromosome 12p is frequently affected by chromosomal gains in our study. In addition, amplification of 12p12, involving the *KRAS* gene, was detected in 10% of cases. This leads us to assume that amplification of 12p seems to play an important role in germinomas. In the last years, several reports already highlighted the importance of chromosome 12p in testicular GCTs, especially of amplification at 12p11.2-p12.1 [28] and describing it as a genetic hallmark of germ cell tumors [29]. Our results strongly confirm these findings for germinomas. On the other hand, previous studies reported the frequency of 12p gain in germinomas as only 36.7% [9] and 36.4% [14], which is a smaller percentage than in our cohort. We propose three explanations for this finding. First, we examined a larger number of germinomas than in the previous studies and maybe therefore obtained more representative data. Second, we examined only tumors of Caucasian patients whereas the previous studies mostly investigated Asian patients. Geographical genetic differences might explain the results. Third, Fukushima et al. [9] described 10% of germinomas as chromosomally stable. In our initial screening, we also identified 4 germinomas of 53 (7.5%) which showed a stable genome in MIP analysis. However, these tumors had a minute amount of tumor cells (< 5%) and mainly consisted of lymphocyte infiltrates contrary to the other samples. For this reason, we suspect that allele frequencies of these tumors are too low to be detected as genetic alterations by MIP analysis. This is also corroborated by the fact that these 4 tumors did not reveal any alterations in the mutation analysis (see below). GISTIC analysis revealed significant CN gains in the *IL10* gene and its receptor genes. Sredni et al. [30] showed that autocrine/paracrine IL10 secretion of stomach adenocarcinoma and glioblastoma multiforme cell lines is essential for tumor cell proliferation and inhibition of IL10 leads to decreased cell proliferation. Future studies might show if germinoma proliferation also depends on IL10.

MIP analysis also detected amplifications at 4q12 and 11p15.2, affecting the *KIT* and *RRAS-2* loci in one case each. As described before, physiological primordial germ cell growth is dependent on *KIT* expression and iGCTs show pathological activation of c-KIT and downstream signaling components. For this reason, we statistically examined the *KIT* and *RAS* family member mutational status in our cohort. We found genetic alterations in 17.3% of germinomas for *KIT* and 34.6% for *RAS* whereas Fukushima et al. [9] detected *c-Kit* mutations in 40% and *RAS* mutations in 20%, Wang et al. [14] 25% for *KIT* and 19% for *RAS* and recently Ichimura et al. [31] found *c-Kit* mutated in 40% and *RAS* mutated in 19% of germinomas. In summary, our overall fraction of alterations in *KIT* and *RAS* is similar to the previous studies. However we found a higher percentage of alterations in *RAS* compared to *KIT*. This difference in the distribution between our cohort and the three collectives above might be due to the ethnicity of the patients leading to geographically different frequencies of genetic alterations in germinomas. Remarkably, all alterations in *KIT* and *RAS* were mutually exclusive genetic events in our cohort. Fukushima et al. [9], Wang et al. [14] and Ichimura et al. [31], who found 97% of germinomas having mutually exclusive mutations in *KIT* or *RAS*, confirmed this finding, which strongly suggests that an alteration in one signal component is sufficient to trigger tumorigenesis. Moreover, in our series we identified *RRAS-2* as a new player in germinoma pathogenesis, which we found alterated in 14.6% of cases.

Furthermore, we discovered activation of the Akt/mTOR pathway by immunohistochemistry in 94.4% and upregulation of ERK pathway in 88.5% of germinomas. Activation of both pathways was detected in 83.3%. Interestingly, pS6 was found to be negative in 57.4% of cases indiacting that this component is not the preferentially phosphorylated target of mTOR in germinomas. Indeed, in other cell types such as cerebellar granule neuron precursors it was shown that mTOR is able to induce eIF4E, which is essential for proliferation, and simultaneously suppresses S6 activity, which is needed for cell cycle exit [32]. Therefore, S6 is not necessary for cell proliferation in cerebellar granule neuron precursors. The main mTOR targets in germinomas and their functional roles have to be identified in future studies.

These findings indicate that activation of Akt/mTOR and ERK pathways are general phenomena in germinomas. Therefore, we suspect that the 43.7% of germinomas with no genetic alterations in our study might have alteration in other components of these pathways besides *KIT* and *RAS*. This is supported by Ichimura et al. [31] who found 7% of germinomas mutated for *MTOR* and further tumors mutated in genes of these two pathways.

Current therapy for intracranial germinomas achieves excellent long term survival, albeit at the cost of some morbidity. Targeted inhibition of the ERK and Akt/mTOR pathways might be an option for patients with recurrent tumors of whose tumors fail to respond to first line therapy. Recently, small molecule inhibitors of Akt were tested at different levels of clinical trials [33]. Phase 1 clinical trials showed positive results for the oral Akt inhibitor afuresertib [34]. In addition, *in vitro* studies with the ERK inhibitor SCH772984 in *RAS* mutant cells exhibited promising results [35]. Ichimura et al. [31] showed that mTor mutant cells which were treated with the mTor inhibitor pp242 showed dose-dependent downregulation of the Akt/mTor pathway. Combining ERK and Akt/mTOR inhibitors might be a therapeutic option for patients with recurrences that could be related to over compensatory hyperactivation of the parallel signal pathway, when only one pathway was inhibited before. However, the dependence of tumor cells on these pathways has to be proven when tumor models become available and further investigations are required to examine targeted therapy options for germinomas.

## MATERIALS AND METHODS

### Tumor specimen

The study includes 54 histologically verified pure germinomas and 1 mixed intracranial germ cell tumor which contained large germinoma parts and some teratoma components. Tumor specimens were retrieved from the archives of the Institute of Neuropathology, University of Bonn Medical Center (Bonn, Germany), of the DGNN German Brain Tumor Reference Center (Bonn, Germany) and the German Paediatric Tumor Registry (Kiel, Germany). All tumors were diagnosed according to the WHO classification of tumors of the central nervous system [36] using histological and immunohistochemical methods.

### DNA extraction

Hematotoxylin-eosin (H&E) –stained sections of each case were examined carefully before DNA extraction. DNA from 53 formalin-fixed paraffin-embedded (FFPE) tumors was extracted using the QIAamp DNA Mini Tissue Kit (Qiagen GmbH; Düsseldorf, Germany) according to the manufacturer's protocol. All tissue samples were used in an anonymous manner, as approved by the ethics committee at the University of Bonn Medical Center (Bonn, Germany).

### Molecular inversion profiling

To identify copy number gains and losses, we used an MIP array using OncoScan FFPE Express 330K Platform Version 2 (Affymetrix, Santa Clara, CA, USA). The molecular inversion probe assay of 53 germinoma samples was performed as already described [37]. By using Nexus Copy Number 7.0 Discovery Edition software (BioDiscovery, El Segundo, CA, USA) the raw MIP data file was analyzed. To make copy number and loss of heterozygosity calls, BioDiscovery's SNP-FASST2-Segmentation algorithm was used. Genomic Identification of Significant Targets in Cancer (GISTIC) analysis was used to distinguish significant chromosomal aberrations from random background [38].

### *KIT* mutation analysis

52 tumors were screened for mutations in *KIT* exons 11, 13, 17 and 18 by direct sequencing. Therefore, polymerase-chain-reaction-products were purified using a PCR purification kit (Qiagen) and sent to a commercial sequencing service (Eurofins MWG Operon, Ebersberg, Germany), where direct sequencing of DNA was performed in duplicate (forward and reverse) using 30 ng of PCR products.

### Pyrosequencing analysis for mutation hot spots of *NRAS*, *KRAS*, *HRAS* and *RRAS-2*

52 specimens were analyzed for hot spot mutations in the RAS genes *NRAS*, *KRAS*, *HRAS* and *RRAS-2*. PCR amplification primers flank the homologous hotspot regions of these genes. For the pyrosequencing reaction, single-stranded DNA templates were immobilized on streptavidin-coated Sepharose high-performance beads (GE Healthcare, Uppsala, Sweden) using the PSQ Vacuum Prep Tool and Vacuum Prep Worktable (Biotage, Uppsala, Sweden), according to the manufacturer's instructions. Then DNA was incubated at 80°C for two minutes and allowed to anneal to 0.4 mmol/L sequencing primer at room temperature. Pyrosequencing was performed using PyroGold Reagents according to the manufacturer's protocol. Positive and negative controls were used to compare results. Pyrograms were analyzed by PyroMark Q24 software (Biotage), using the allele quantification (AQ) software to determine the percentage of mutant versus wild type alleles according to percentage relative peak height. A sequential nucleotide dispensation protocol was used, reflecting the expected order of nucleotide incorporation and the potential base change within the first, second or third position of the hot spot codons G12, G13 and Q61 of *HRAS*, *NRAS* and *KRAS*. In the same manner codons G23, G24 and Q72 of *RRAS-2* were examined. Peak heights are proportional to the number of nucleotides that are incorporated with each dispensation.

### Immunohistochemistry

Paraffin-embedded tissue samples were divided into serial sections of 4 μm. Positively charged slides were cut, air dried overnight at 37°C, deparaffinized in xylene and rehydrated in a degraded alcohol sequence. Slides were then microwaved in 10 mmol/L citrate buffer (pH 6.0) for antigen retrieval, followed by incubation in 3% hydrogen peroxide for 5 minutes at room temperature to block the activity of endogenous peroxidase and in blocking solution (CSA II Kit; Dako, Glostrup, Denmark). Tumors were incubated overnight with primary antibodies [pAkt Ser473, 1:200, rabbit monoclonal antibody (736E11, Cell Signaling Technology, Leiden, Netherlands); pERK Thr202/Tyr204, 1:200, rabbit monoclonal antibody (20G11, Cell Signaling Technology); pS6 Ser235/236, 1:200, rabbit monoclonal antibody (91B2, Cell Signaling Technology); pmTOR Ser2448, 1:50, rabbit monoclonal antibody (49F9, Cell Signaling Technology); 5-Methylcytosine, 1:8000, mouse monoclonal antibody (BI-MECY, IgG1, Eurogentec, Seraing, Belgium)]. Sections were washed in Tris-buffered saline with Tween 20. Visualization of bound antibodies was achieved by the CSA system embodies technology (CSA II, Biotin-Free catalyzed Amplification System; Dako). Slides were counterstained with hematoxylin and dehydrated in a graded alcohol sequence and mounted in Richard-Allan Scientific Cytoseal XYL (Thermo Scientific, Waltham, MA).

Two authors blinded for clinical and genomic data evaluated the percentage of stained cells and scored the samples for staining intensity (0 = negative, 1 = weak intensity, 2 = moderate intensity, 3 = strong intensity). The results were used to calculate a score between 0 and 300 by multiplying the percentage of stained cells by intensity, and tumor samples were classified into negative staining (score 0-9), weak staining (score 10-49), moderate staining (score 50-199) and strong staining (score 200-300).

### Statistical analysis

Significantly overrepresented factor values in a particular factor group were identified using the one-tailed Fisher's Exact test at a p-value of 0.05. The log-rank test statistic [39] was used to identify regions yielding a high degree of survival prediction. p-values were calculated based on a Chi-Squared test and p-value ≤ 0.05 were considered significant. To compare survival times, Kaplan-Meier curves were generated. The p-values were computed using the log-rank test [39].
